# Identification of dilated cardiomyopathy‐linked key genes by bioinformatics methods and evaluating the impact of tannic acid and monosodium glutamate in rats

**DOI:** 10.1002/bab.2670

**Published:** 2024-09-25

**Authors:** Habibe Karadas, Hilal Tosun, Hamid Ceylan

**Affiliations:** ^1^ Department of Molecular Biology and Genetics, Faculty of Science Atatürk University Erzurum Turkey

**Keywords:** bioinformatics, dilated cardiomyopathy, heart, monosodium glutamate, tannic acid

## Abstract

Dilated cardiomyopathy (DCM) is the most common type of myocardial dysfunction, affecting mostly young adults, but its therapeutic diagnosis and biomarkers for prognosis are lacking. This study aimed to investigate the possible effect of the common food additive monosodium glutamate (MSG) and tannic acid (TA), a phenolic compound, on the key molecular actors responsible for DCM. DCM‐related publicly available microarray datasets (GSE120895, GSE17800, and GSE19303) were downloaded from the comprehensive Gene Expression Omnibus (GEO) database, and analyzed to identify differentially expressed genes (DEGs). By integrating DEGs and gene‐disease validity curation results, overlapping genes were screened and identified as hub genes. Protein‐protein interaction (PPI) network and ontology analysis were performed to make sense of the identified biological data. Finally, mRNA expression changes of identified hub genes in the heart tissues of rats treated with MSG and TA were measured by the qPCR method. Six upregulated (*IGF1, TTN, ACTB, LMNA, EDN1*, and *NPPB*) DEGs were identified between the DCM and healthy control samples as the hub genes. qPCR results revealed that the mRNA levels of these genes involved in DCM development increased significantly in rat heart tissues exposed to MSG. In contrast, this increase was remarkably alleviated by TA treatment. Our results provide new insights into critical molecular mechanisms that should be focused on in future DCM studies. Moreover, MSG may play a critical role in DCM formation, and TA may be used as a promising therapeutic agent in DCM.

**Abbreviations**
GEO: Gene Expression Omnibus
GEPIA: Gene expression profiling interactive analysis
KM‐plotter: Kaplan‐Meier plotter
WHO: World Health Organization
GLOBOCAN: Global Cancer Incidence, Mortality and Prevalence
DAVID: The Database for Annotation, Visualization, and Integrated Discovery
DEGs: Differentially expressed genes
PPI: The protein‐protein interaction
STRING: The search tool for the retrieval of interacting genes
GO: Gene ontology
KEGG: Kyoto encyclopedia of genes and genome

## 1 INTRODUCTION

Cardiovascular diseases (CVDs) are a public health problem, whose prevalence increases with the global population and accounts for approximately 35% of deaths worldwide.^1^, ^2^ Dilated cardiomyopathy (DCM), a common type of cardiomyopathy, is a disease of the enlargement and dilation of the heart muscle. The exact cause of this condition was initially unknown, but many risk factors have been associated with DCM, including chemotherapy drugs,^3^ physical inactivity,^4^ excessive alcohol and cigarette use,^5^ and individual lifestyle behaviors such as an unhealthy diet.^6^ Nutrition, although an inevitable long‐term environmental factor, is defined as a modifiable risk for DCM.^7^ Numerous studies have revealed that dietary nutrients or metabolites have the potential to trigger mechanisms that can lead to inflammation,^8^ oxidative stress,^9^ and an increased risk of DCM through their potentially toxic effects on the heart. In addition to predisposing the development of DCM, prominent clinical knowledge has revealed a strong relationship between an unhealthy diet and a response to CVD treatment.

As it is known, in recent years, human beings' dietary habits have changed greatly along with their lifestyle.^10^ Today, when globalization has increased considerably, cultural evolution has also accelerated and has greatly changed the nutritional habits of societies. Along with these sociological developments, the demand for processed and ready‐made foods has increased since the second half of the last century.^11^ Nowadays, the use of food additives, which are used to increase the appearance, taste, and odor of commercial foods, as well as to extend their shelf life, is increasing significantly.^12^ Monosodium glutamate (MSG), a commonly consumed additive, is the sodium salt of glutamic acid that adds umami flavor, especially to processed foods.^13^ Although the World Health Organization (WHO) and the Food and Drug Administration (FDA) consider MSG in the safe food ingredient category, studies associate MSG with different organ toxicities.^14^ Additionally, MSG use has been reported to be linked to health problems such as cancer, dementia, obesity, and CVDs, which are classified as noncommunicable diseases (NCDs) by WHO.^15^ Although 16.0 mg/kg bodyweight is considered a no‐observed‐adverse‐effect level (NOAEL) for MSG consumption,^16^ it has been reported that MSG exposure at lower concentrations may lead to various anomalies that affect the quality of life, such as obesity,^17^ liver cancer,^18^ renal toxicity,^19^ and cardiotoxicity.^20^ Therefore, elucidating the dietary components that negatively affect cellular physiology in pathologies such as CVDs and the action mechanisms of these factors may advance the development of effective therapeutic intervention approaches.^21^, ^22^, ^23^

Recently, bioactive natural compounds have gained attention in combating complex diseases such as cancer, obesity, and CVDs.^24^ These naturally occurring compounds tend to have fewer side effects compared to synthetic pharmaceuticals. Studies have shown that dietary phytochemicals could be cost‐effective and less toxic options for preventing or slowing disease progression in various conditions, including DCM.^25^ Tannic acid (TA), a natural polyphenol, possesses anticarcinogenic, antimutagenic, anti‐inflammatory, and antioxidant properties.^26^, ^27^ These properties make TA a promising candidate against myocardial infarction, renal failure, and cancer.

Therefore, this work focused on identifying DCM‐associated hub genes using integrated bioinformatics analysis of publicly available Gene Expression Omnibus (GEO) microarray datasets. Moreover, the present study allows examination of the effect of MSG exposure and TA treatment on the regulation of DCM‐associated hub genes in experimental rat models.

## 2 MATERIALS AND METHODS

### 2.1 Microarray data profiles

Three DCM‐associated independent publicly available datasets were extracted from the GEO database (https://www.ncbi.nlm.nih.gov/geo/).^28^ GSE120895 consists of endomyocardial biopsies (EMBs) of 47 DCM patients and eight healthy patient heart samples.^29^ GSE17800 consists of EMBs of 40 DCM patients, as well as eight healthy controls.^30^ GSE19303 consists of EMBs of 73 DCM patients and eight healthy heart samples as control.^31^ All expression profiling arrays were generated using the [HG‐U133_Plus_2] Affymetrix Human Genome U133 Plus 2.0 platform (GPL570).

### 2.2 Data pre‐processing and screening for DEGs

Differentially expressed genes (DEGs) between EMBs of DCM patients and healthy individuals’ samples were screened and analyzed using GEO2R (http://www.ncbi.nlm.gov/geo2r). *p*‐Value less than 0.05 and a |log2FC| ≥ 0.5 were defined as the cut‐off criteria. Common genes shared in all datasets were determined by generating a Venn diagram using a list comparator (http://www.molbiotools.com/listcompare.html).

### 2.3 Disease gene curation of DEGs

For DCM‐related gene curation, identified DEGs were text‐mined using a database of gene‐disease associations (DisGeNet; https://www.disgenet.org/).^32^ The human disease database (MalaCards; https://www.malacards.org/)^33^ and Free Biomarker Database (BIONDA; http://bionda.mpc.ruhr‐uni‐bochum.de/start.php).^34^ Finally, a Venn diagram summarizing the overlapping DEGs across all datasets and curated candidate genes involved in DCM was generated using a list comparator (http://www.molbiotools.com/listcompare.html).

### 2.4 Protein‐protein interaction

Protein‐protein interaction (PPI) network evaluation was carried out to examine the interaction of each protein encoded by the identified common genes.^35^ The network was mapped by using the Search Tool for the Retrieval of Interacting Genes (STRING; https://string‐db.org/) online tool^36^ under the default settings and visualized by using the Cytoscape (version 3.9.1) software.^37^

### 2.5 Functional and pathway enrichment analysis of hub genes

To identify the Gene Ontology (GO), KEGG (Kyoto Encyclopedia of Genes and Genome) pathway enrichment, and disease relations of the shortlisted DEGs, the ToppFun module of ToppGene Suite (https://toppgene.cchmc.org/enrichment.jsp)^38^ was used.

### 2.6 Animals and experimental design

A total of 24 male rats (*Rattus norvegicus*, Sprague‐Dawley, male, 180 ± 10 g) were purchased from the Atatürk University Medical Experimental Application and Research Center and randomly divided into four (control and three experimental, six rats in each) groups. The control (CON) group was treated with saline via oral gavage daily. TA group rats received 50 mg/kg TA via oral gavage daily.^39^, ^40^ MSG group rats received 2 g/kg MSG via oral gavage daily.^41^, ^42^ The MSG and TA were administered simultaneously to the combined group. All animals were housed at a 12‐h/12‐h light/dark cycle, 22°C ± 3°C temperature, 50%–60% humidity, and fed with standard pellet and water ad libitum. At the end of the 3 weeks (21st day), all rats were euthanized under ketamine/xylazine (3:1) anesthesia. Promptly after, the heart tissues were taken and kept at −80°C for further studies. All of the experimental procedures were performed under the National Research Council's Guide for the Care and Use of Laboratory Animals and were approved by the Atatürk University Local Ethics Council for Animal Experiments (Protocol No: 2021‐3/63).

### 2.7 RNA extraction and cDNA synthesis

Total RNA was isolated from rat heart tissues using a commercial extraction kit (Biorad) according to the manufacturer's manual. RNA concentrations and purity were quantified and verified by spectrophotometer (Thermo Scientific, Multiskan GO). Complementary DNA (cDNA) was synthesized from 1 µg total RNA using a commercial cDNA synthesis kit (Biorad) following the manufacturer's protocol.

### 2.8 Quantitative real‐time PCR

Quantitative changes in mRNA expression of cardiac damage marker genes and hub genes were measured using a SYBR Green‐based qPCR assay. All gene‐specific primer sets used in quantitative real‐time PCR (qPCR) analysis (Table 1) were designed and verified as previously described.^15^ Amplification reactions were carried out using SsoAdvanced Universal SYBR Green Supermix (Biorad) in a thermal cycler (Rotor‐Gene Q, Qiagen) according to the manufacturer's protocol. *Gapdh* (glyceraldehyde 3‐phosphate dehydrogenase, NM_017008.3) was used as housekeeping control. The comparative ΔΔCt method^43^ was used for the relative quantification.

**TABLE 1:** Sequences of primer sets used in qPCR.

Gene symbol	Accession ID	Sequence	*T*_m_ (°C)
*β‐Mhc*	NM_017240.2	F: 5ʹ‐TTGATGTGCTGGGCTTCAC‐3ʹ	60.42
		R: 5ʹ‐CTCCTCCCTCTGCTTCTGTT‐3ʹ	59.98
*Anf*	M27498.1	F: 5ʹ‐AGAGAGTGAGCCGAGACAGC‐3ʹ	59.89
		R: 5ʹ‐AGCCCTTGGTGATGGAGAA‐3ʹ	60.61
*Bnp*	M25297.1	F: 5ʹ‐ACAAGAGAGAGCAGGACACC‐3ʹ	59.12
		R: 5ʹ‐AAAGCAGGAGCAGAATCATC‐3ʹ	59.20
*Actb*	NM_031144.3	F: 5ʹ‐TGTGGATTGGTGGCTCTATC‐3ʹ	59.78
		R: 5ʹ‐AGAAAGGGTGTAAAACGCAG‐3ʹ	60.23
*Igf1*	NM_178866.4	F: 5‐TTACTTCAACAAGCCCACAGG‐3	60.15
		R: 5‐ATCCACAATGCCCGTCTG‐3	60.50
*Ttn*	XM_039106393.1	F: 5‐GCGCATACAGAACATCGTGG‐3	59.70
		R: 5‐TCAGCTCTGTCGGTCCTTTG‐3	59.97
*Lmna*	NM_001002016.2	F: 5‐TAGAGGGTGTGGTGATTGGTG‐3	60.82
		R: 5‐AGATGGGAAGTGGGATGAAG‐3	60.86
*Edn1*	NM_012548.2	F: 5‐TTGCCTCTTCTTGCTGTCTG‐3	59.31
		R: 5‐ATCTCCTGGCTCTCTGGATG‐3	59.36
*Gapdh*	NM_017008.3	F: 5ʹ‐AAACCCATCACCATCTTCCA‐3ʹ	60.17
		R: 5ʹ‐ATACTCAGCACCAGCATCACC‐3ʹ	60.16

### 2.9 Statistical analysis

Statistical comparison of data obtained from measurements made in triplicate (for each animal and sample) was evaluated with one‐way analysis of variance (ANOVA) and Tukey's post hoc test using Prism (GraphPad Software) software. The statistically significant differences are presented as follows: ^ns^ *p* > 0.05 (not significant, compared to the control group); *p < 0.05 (significant); **p < 0.01 (very significant); *** or ****p < 0.001 or.0001 (extremely significant).

## 3 RESULTS

### 3.1 Identification of common DEGs of DCM

**FIGURE 1:**
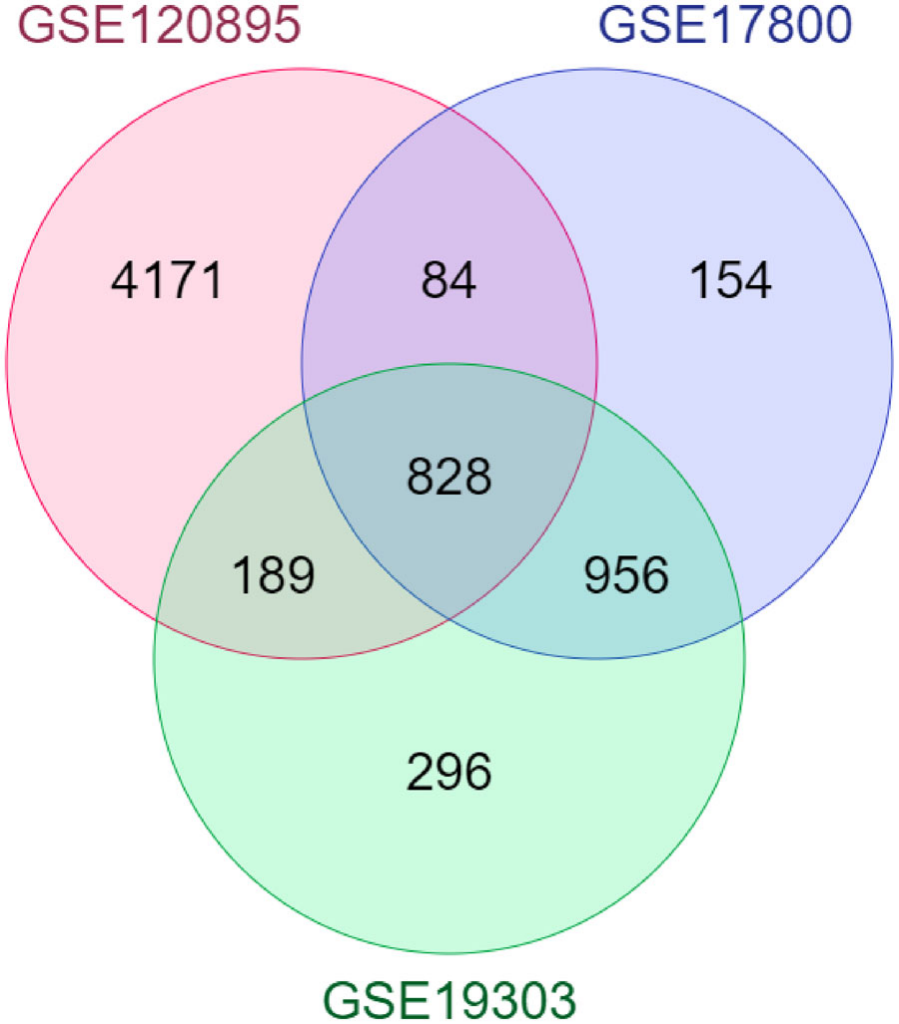
Venn diagram of the differentially expressed genes (DEGs) between the integrated three Gene Expression Omnibus (GEO) datasets.

Upregulated and downregulated DEGs were screened from GSE120895, GSE17800, and GSE19303, respectively, by GEO2R. Subsequently, we identified and selected for further analysis 828 shared upregulated genes related to DCM in three datasets using a Venn diagram (Figure 1). No shared downregulated gene was detected in the three datasets used.

### 3.2 DCM‐related gene curation for DEGs

**FIGURE 2:**
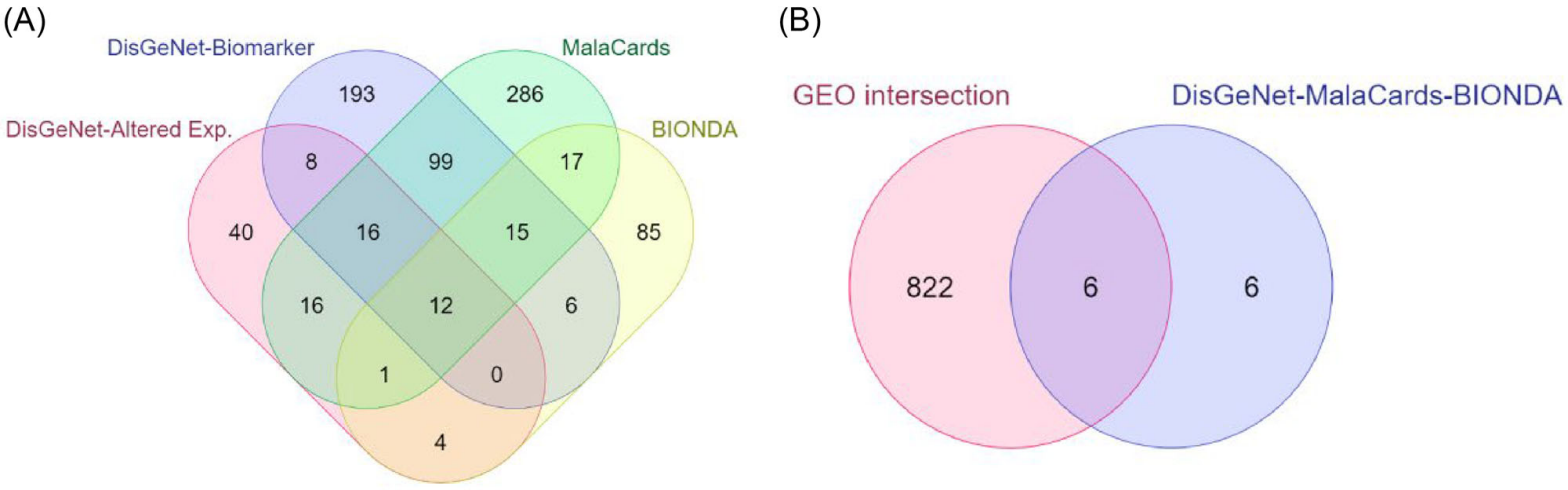
Venn diagrams of the disease‐gene curation. (A) Venn diagram of the curation databases. (B) Venn diagram of common genes shared in both Gene Expression Omnibus (GEO) datasets and curation databases.

To test the validity of the extracted DEGs from GEO datasets, the gold benchmark databases, including DisGeNet, BIONDA, and MalaCards, were used. Altered expression and biomarker association type ontology results were extracted from DisGeNet to indicate the associations of DEGs with the disease phenotype and their role in the etiology of the disease, respectively. Mining results revealed 36 DCM‐associated genes in DisGeNet, 140 in BIONDA, and 462 in MalaCards (Figure 2A). Twelve genes were found to be common to these three databases. Finally, six genes (*IGF1*, *TTN*, *ACTB*, *LMNA*, *EDN1*, and *NPPB*) were found to be in common between both the DEGs represented in the GEO datasets and the results obtained from the curation databases (Figure 2B) and identified as hub genes for DCM.

### 3.3 PPI network analysis

**FIGURE 3:**
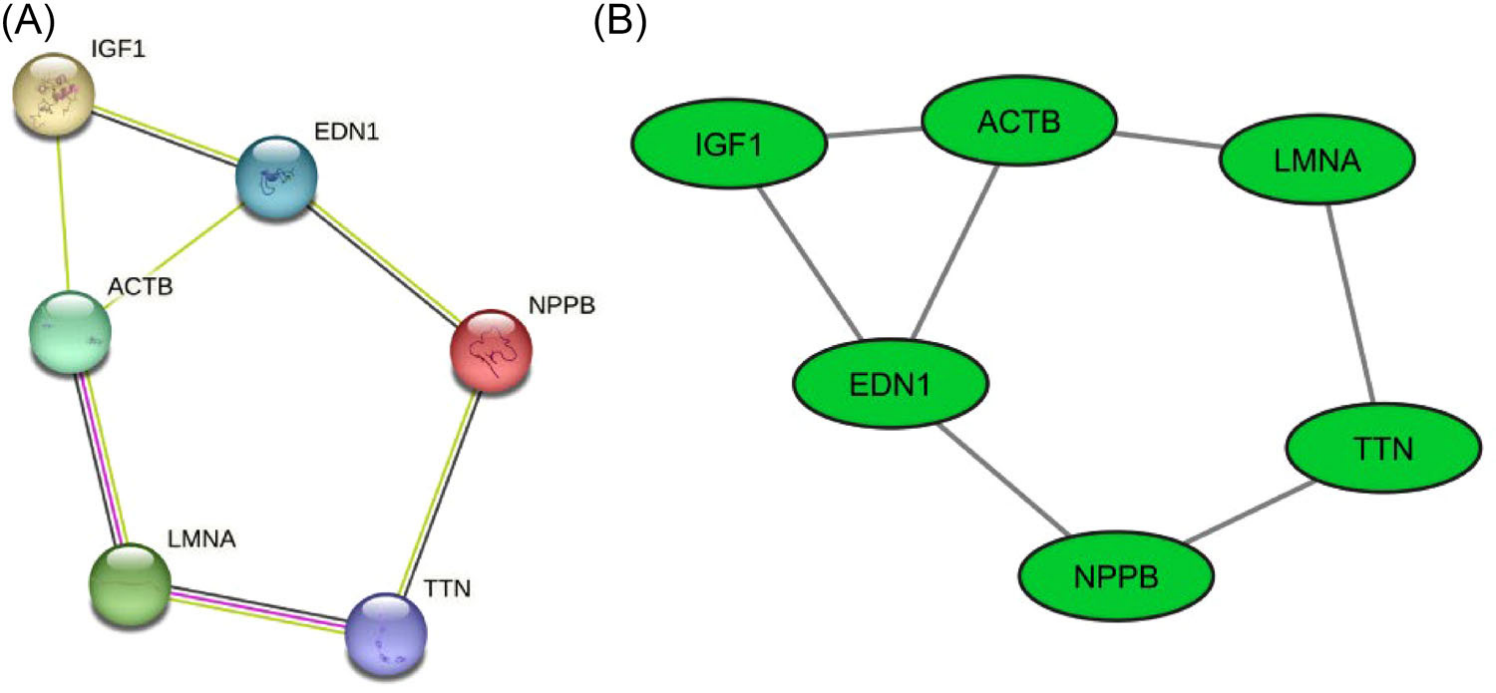
Protein–protein interaction (PPI) network of dilated cardiomyopathy (DCM)‐related hub genes. (A) STRING network for six hub genes. (B) PPI network of hub genes generated with Cytoscape. Edges represent the PPI and the green nodes represent upregulated genes.

A PPI network was formed to examine the interaction between genes whose expression was significantly altered in heart tissues obtained from patients diagnosed with DCM compared to healthy heart tissue samples. PPI was first mapped STRING (Figure 3A) and then visualized by using the Cytoscape (Figure 3B). Network analysis of hub genes revealed six nodes and seven edges in the PPI network.

### 3.4 Enrichment analysis of overlapping DEGs

To further explain potential functions associated with the six DEGs of DCM, we conducted GO and pathway (KEGG) enrichment analysis. DEGs were classified into molecular functions, biological processes, and cellular components. The pathway analysis results indicated that hub genes were largely involved in cardiac abnormalities including cardiomyopathy (Table S1). In the next step, the transcriptional changes of the hub genes were tested in rat heart tissue after MSG exposure and TA treatment.

### 3.5 qPCR analysis

**FIGURE 4:**
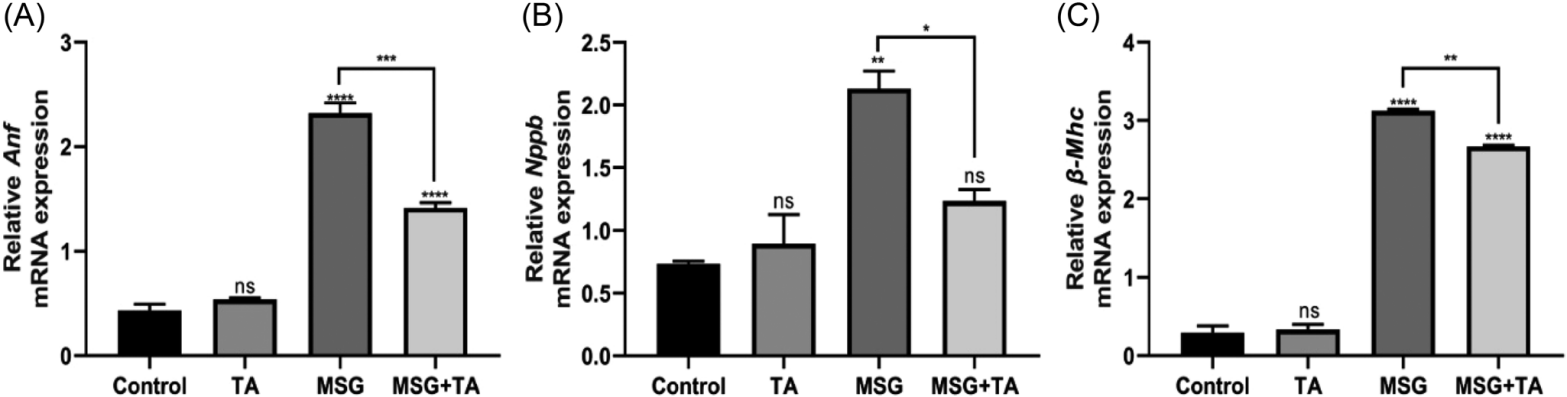
mRNA expression profiling of cardiac damage markers in the heart tissues of the control versus treated groups. (A) The relative mRNA expression levels of *Anf*. (B) The relative mRNA expression levels of *Nppb*. (C) The relative mRNA expression levels of *β‐Mhc*.

First, to test whether MSG causes damage to the heart tissue and whether TA reduces the damage, mRNA expression levels of cardiac damage marker genes were measured by qPCR. qPCR analysis results demonstrated that all cardiac damage‐related marker genes (atrial natriuretic peptide prohormone, *Anf*, also known as *Nppa*, natriuretic peptide precursor B; *Nppb*, also known as *Bnp*, and myosin, heavy chain 7, cardiac muscle, beta; *β‐Mhc*) expressions were significantly upregulated following 21 days of exposure to MSG. However, abnormal regulation of these genes was reverted with TA treatment (Figure 4A–C). These findings clearly show that MSG may cause cardiac anomalies, but TA may help reduce the negative prognosis caused by MSG.

**FIGURE 5:**
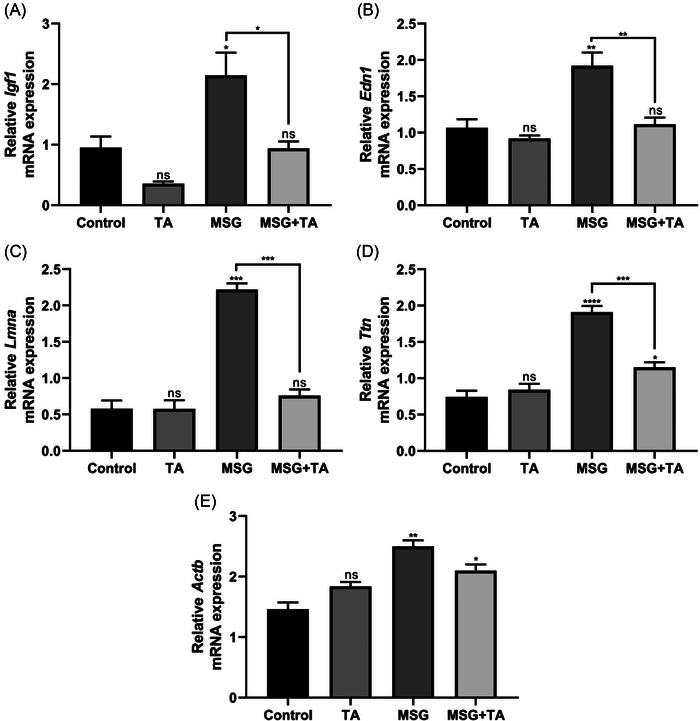
mRNA expression profiling of hub genes in the heart tissues of the control versus treated groups. (A) Relative expression of *Igf1*. (B) Relative expression of *Edn1*. (C) Relative expression of *Lmna*. (D) Relative expression of *Ttn*. (E) Relative expression of *Actb*.

We have also explored the mRNA expression alterations of identified hub genes (*Igf1*, *Edn1, Lmna, Ttn*, *Actb*, and *Nppb*) in all experimental rat groups. It was observed that the transcriptional activities of insulin‐like growth factor 1 (*Igf1*), endothelin 1 (*Edn1*), lamin A/C (Lmna), titin (*Ttn*), and actin beta (*Actb*) were significantly upregulated after MSG exposure. However, it was observed that simultaneous TA treatment with MSG significantly suppressed the mRNA expression of these genes in heart tissues (Figure 5A–E). *Nppb* is not repeated here, because it is also among the cardiac damage markers (Figure 4B). The present findings strongly suggest that MSG can lead to DCM formation by aggressively inducing mRNA expressions of DCM‐related hub genes, but this increase is attenuated by TA.

## 4 DISCUSSION

CVDs are among the NCDs with a high mortality rate that cause the majority of annual deaths worldwide.^44^, ^45^, ^46^, ^47^ For this reason, diagnosis and treatment studies for CVDs attract the intense attention of researchers today. Identifying key biomarkers associated with CVDs, which have complex and not fully understood pathologies, is crucial for preventing disease progression and enhancing patients’ quality of life.^48^, ^49^ Bioinformatics approaches are important in investigating genes that stand out in NCDs such as CVD due to their polygenic nature and multiple gene expression dysregulation.^50^ In this study, not only the analysis of publicly available GEO datasets identified candidate hub genes involved in DCM, an important type of CVDs, but also the diet‐related behaviors of these genes were examined in experimental rat models. In this study, the three datasets GSE120895, GSE17800, and GSE19303 were selected from the GEO database, and DCM‐associated 828 upregulated DEGs were identified. Moreover, to identify critical hub genes by narrowing the number of target genes, disease‐gene curation data were scanned from disease databases and six genes (*IGF1, TTN, ACTB, LMNA, EDN1*, and *NPPB*) were identified as hub genes.

Insulin‐like growth factor 1 (encoded by the *IGF1* gene) is a growth hormone implicated in the regulation of cardiac growth and stimulation of myocardial contraction.^51^ Clinical studies have outlined that overexpressed IGF‐1 is correlated with the development of heart failure and elevated mortality. Interestingly, other studies have revealed that prolonged IGF‐1 exposure is also linked to cancer development.^52^, ^53^ Several epidemiological and in vivo studies show that IGF1 directly affects aging, an independent risk factor for CVDs, through different complex mechanisms. It has been shown in studies conducted with experimental animal models that reduced IGF1 signaling extends lifespan.^54^ In another study conducted with cultured neonatal rat cardiomyocytes, it was determined that IGF1 increased mRNA levels of *MLC‐2* and troponin associated with infant‐onset myopathy and hypertrophic cardiomyopathy.^55^ All these findings reveal that IGF1 activity is associated with CVDs in humans. In this work, results showed that *IGF1* was upregulated in the heart tissue of MSG‐exposed rats compared to the controls. However, it was noticed that simultaneous TA treatment with MSG significantly suppressed *IGF1* mRNA expression, which increased with MSG.

Endothelin 1 (EDN1) is a potent vasoconstrictor that plays a critical role in maintaining blood pressure by regulating salt and water homeostasis.^56^ Previous studies have shown that individuals with DCM have increased plasma EDN1 level^57^ and increased *EDN1* mRNA transcription in heart tissue.^58^ Increased EDN1 peptide concentration activates the local cardiac endothelin system, which may lead to the narrowing of arteries and vessels. This condition reduces coronary blood flow and increases the left ventricular load, which deteriorates the contractility of the heart, causing DCM.^59^ In addition, studies have shown that overexpression of *EDN1* expression can assist in the upregulation of inflammatory cytokines that trigger myocarditis and lead to heart failure.^60^

It is known that cytoskeleton‐related proteins are essential in the pathogenesis and pathophysiology of DCM and other cardiomyopathies.^61^ Expression shifts of these actors may also affect their downstream targets, leading to increased myocyte degeneration and fibrosis, ultimately resulting in chronic cardiac anomalies such as DCM.^62^ TTN (titin), a giant structural and regulatory protein critical for cardiac contraction and relaxation, is particularly involved in cell signaling and elasticity of striated muscle.^63^ *TTN* expressed in adult cardiac muscle tissue is spliced into two major titin isoforms, N2BA (longer) and N2B (shorter). Recent studies showed increased expression of N2BA:N2B titin protein isoforms in failing human DCM heart samples.^64^ Another study in guinea pigs showed that *Ttn* transcript expression was increased in cardiomyopathy models.^65^ LMNA (Lamin A/C) is another mechanoprotective protein that is ubiquitously expressed in mammalian somatic cells and may limit progressive myocyte loss.^66^ However, abnormal activation of LMNA, which is commonly mutated in DCM and can lead to a worst prognosis and sudden death, has been documented in human subjects and various experimental models of cardiomyopathies.^67^, ^68^ Therefore, cardiomyopathy resulting from alterations in type A lamins has been referred to as LMNA cardiomyopathy.^69^ This knowledge implies the involvement of TTN, together with LMNA, in the progression of DCM.

Actin beta (encoded by *ACTB*), an important unit of the cytoplasmic cytoskeleton, is a highly conserved protein involved in intercellular signaling, cell motility, and contraction.^70^ Increased contractility of cardiomyocytes assists the pumping capacity, especially in the postnatal period.^71^ However, abnormal dysregulation of cardiomyocyte contractility can lead to hypertrophic cardiomyopathy (HCM) and DCM.^67^ Gene expression profiling studies in different types of cardiomyopathy models have reported that various cytoskeletal proteins, such as β‐actin, are overexpressed, and promote cell spreading and myocyte contractility.^72^, ^73^ These key findings highlight the role and dynamics of β‐actin in cardiomyopathy stimulation.

NPPB (brain natriuretic peptide or BNP) is a protein that acts as a cardiac hormone involved in cardiovascular homeostasis.^74^ As it is strongly stimulated by cardiac stress and heart failure in atrial and ventricular heart muscle cells, it is a common and reliable marker gene used, together with ANF, in monitoring HF‐related diseases, including cardiomyopathy.^75^ It has been previously reported that transcript levels of *BNP* and the genes examined in this study (*Anf*, *βMhc*, and *Actn*) are higher in the failing heart than in the healthy heart samples.^76^ Microarray‐based gene expression profiling studies also revealed significantly increased expression of NPPB in heart samples from both male and female patients with end‐stage and new‐onset DCM.^77^, ^78^ Moreover, the RNA‐seq approach to identify DEG signatures in ischemic heart disease (ISCH) and DCM heart samples discovered that NPPB overexpressed in both conditions.^79^ The reported data suggest that accurate modulation of NPPB provides a restorative possibility for DCM.

Our study provides evidence that MSG plays a role in DCM by causing alterations in key DCM‐related genes responsible for cytoskeleton rearrangement, myocardial contractility, and intercellular signaling. However, it appears that the potential mechanisms underlying MSG‐induced DCM development can be accurately stimulated by TA treatment. Although our findings suggest an alternative approach to DCM therapy, there are still some limitations. Clinical trials and also further experimental analysis are required for long‐term validation. In addition, the possible downstream mechanisms and molecular drivers affected by hub genes in DCM need to be examined in further experiments.

## 5 CONCLUSION

In conclusion, this study demonstrates that hub genes and pathways found through a comprehensive integrated bioinformatics analysis may be associated with DCM progression and may be potential biomarkers and therapeutic targets to focus on in future studies. Furthermore, the expression of these six DCM‐related hub genes was confirmed at the mRNA level in the heart tissue of rats exposed to MSG and also treated with TA. The findings of this study need further in vivo experimental validation but can be argued to be valuable promising arguments for combating DCM.

## AUTHOR CONTRIBUTIONS

**Hamid Ceylan**: Conceptualization and design; data curation; formal analysis; writing—original draft. **Habibe Karadas** and **Hilal Tosun**: Data curation; formal analysis; animal care; and experiments. All authors reviewed the results and approved the final version of the manuscript.

## CONFLICT OF INTEREST STATEMENT

The authors declare that there is no conflict of interest with any financial organization or corporation or individual that can inappropriately influence this work.

## FUNDING INFORMATION

This research did not receive any specific grant from funding agencies in the public, commercial, or not‐for‐profit sectors.

## Supporting information




**Table S1** GO (Gene Ontology) and KEGG (Kyoto Encyclopedia of Genes and Genomes) pathway enrichment analysis results of genes found to be differentially expressed (*p*‐value <0.05, |log2FC| ≥ 0.05 and |log2FC| ≤ 0.05). MF: molecular function, BP: biological process, and CC: cellular component.

## Data Availability

The data used and analyzed during the current study are available from the corresponding author upon reasonable request.
