# Supplementary material for: Identification of dilated cardiomyopathy‐linked key genes by bioinformatics methods and evaluating the impact of tannic acid and monosodium glutamate in rats
Source: Biotechnol Appl Biochem. 2024 Sep 25;72(2):377–87. doi: 10.1002/bab.2670 (PMC11975261; doi:10.1002/bab.2670)
Supplement: Supplementary file 1 — Table S1 GO (Gene Ontology) and KEGG (Kyoto Encyclopedia of Genes and Genomes) pathway enrichment analysis results of genes found to be differentially expressed (p‐value <0.05, |log2FC| ≥ 0.05 and |log2FC| ≤ 0.05). MF: molecular function, BP: biological process, and CC: cellular component. [file BAB-72-377-s001.docx]

**Supplementary Table 1**. GO (Gene Ontology) and KEGG (Kyoto Encyclopedia of Genes and Genomes) pathway enrichment analysis results of genes found to be differentially expressed (*p*-value <0.05, |log2FC| ≥ 0.05 and |log2FC| ≤ 0.05). **MF**; Molecular Function, **BP**; Biological Process, and **CC**; Cellular Component.

| Category | ID | Name | p-value | q-value Bonferroni | q-value FDR B&H | q-value FDR B&Y | Hit Count in Query List | Hit Count in Genome | Hit in Query List |
| --- | --- | --- | --- | --- | --- | --- | --- | --- | --- |
| MF | GO:0005179 | hormone activity | 5.873E-6 | 4.463E-4 | 2.385E-4 | 1.172E-3 | 3 | 134 | EDN1,IGF1,NPPB |
| MF | GO:0005200 | structural constituent of cytoskeleton | 6.277E-6 | 4.771E-4 | 2.385E-4 | 1.172E-3 | 3 | 137 | LMNA,TTN,ACTB |
| MF | GO:0031707 | endothelin A receptor binding | 3.014E-4 | 2.291E-2 | 5.206E-3 | 2.558E-2 | 1 | 1 | EDN1 |
| MF | GO:0008613 | diuretic hormone activity | 3.014E-4 | 2.291E-2 | 5.206E-3 | 2.558E-2 | 1 | 1 | NPPB |
| MF | GO:0048018 | receptor ligand activity | 3.897E-4 | 2.962E-2 | 5.206E-3 | 2.558E-2 | 3 | 548 | EDN1,IGF1,NPPB |
| MF | GO:0030546 | signaling receptor activator activity | 4.110E-4 | 3.123E-2 | 5.206E-3 | 2.558E-2 | 3 | 558 | EDN1,IGF1,NPPB |
| MF | GO:0030545 | signaling receptor regulator activity | 5.574E-4 | 4.236E-2 | 6.052E-3 | 2.974E-2 | 3 | 619 | EDN1,IGF1,NPPB |
| MF | GO:0031708 | endothelin B receptor binding | 9.038E-4 | 6.869E-2 | 8.586E-3 | 4.219E-2 | 1 | 3 | EDN1 |
| MF | GO:0031705 | bombesin receptor binding | 1.205E-3 | 9.157E-2 | 1.017E-2 | 5.000E-2 | 1 | 4 | EDN1 |
| MF | GO:0005198 | structural molecule activity | 1.720E-3 | 1.308E-1 | 1.308E-2 | 6.426E-2 | 3 | 911 | LMNA,TTN,ACTB |
| MF | GO:0031433 | telethonin binding | 2.108E-3 | 1.602E-1 | 1.456E-2 | 7.157E-2 | 1 | 7 | TTN |
| MF | GO:0030957 | Tat protein binding | 3.010E-3 | 2.287E-1 | 1.906E-2 | 9.368E-2 | 1 | 10 | ACTB |
| MF | GO:0098973 | structural constituent of postsynaptic actin cytoskeleton | 3.310E-3 | 2.516E-1 | 1.935E-2 | 9.511E-2 | 1 | 11 | ACTB |
| MF | GO:0097493 | structural molecule activity conferring elasticity | 4.212E-3 | 3.201E-1 | 2.286E-2 | 1.123E-1 | 1 | 14 | TTN |
| MF | GO:0051371 | muscle alpha-actinin binding | 4.512E-3 | 3.429E-1 | 2.286E-2 | 1.123E-1 | 1 | 15 | TTN |
| MF | GO:0005159 | insulin-like growth factor receptor binding | 5.412E-3 | 4.113E-1 | 2.571E-2 | 1.263E-1 | 1 | 18 | IGF1 |
| MF | GO:0030297 | transmembrane receptor protein tyrosine kinase activator activity | 6.312E-3 | 4.797E-1 | 2.765E-2 | 1.359E-1 | 1 | 21 | IGF1 |
| MF | GO:0030506 | ankyrin binding | 6.911E-3 | 5.253E-1 | 2.765E-2 | 1.359E-1 | 1 | 23 | TTN |
| MF | GO:0050998 | nitric-oxide synthase binding | 6.911E-3 | 5.253E-1 | 2.765E-2 | 1.359E-1 | 1 | 23 | ACTB |
| MF | GO:0005158 | insulin receptor binding | 9.007E-3 | 6.845E-1 | 3.233E-2 | 1.589E-1 | 1 | 30 | IGF1 |
| MF | GO:0099186 | structural constituent of postsynapse | 9.605E-3 | 7.300E-1 | 3.233E-2 | 1.589E-1 | 1 | 32 | ACTB |
| MF | GO:0051393 | alpha-actinin binding | 9.605E-3 | 7.300E-1 | 3.233E-2 | 1.589E-1 | 1 | 32 | TTN |
| MF | GO:0051427 | hormone receptor binding | 1.020E-2 | 7.754E-1 | 3.233E-2 | 1.589E-1 | 1 | 34 | NPPB |
| MF | GO:0008157 | protein phosphatase 1 binding | 1.110E-2 | 8.435E-1 | 3.233E-2 | 1.589E-1 | 1 | 37 | LMNA |
| MF | GO:0005102 | signaling receptor binding | 1.117E-2 | 8.488E-1 | 3.233E-2 | 1.589E-1 | 3 | 1756 | EDN1,IGF1,NPPB |

| Category | ID | Name | p-value | q-value Bonferroni | q-value FDR B&H | q-value FDR B&Y | Hit Count in Query List | Hit Count in Genome | Hit in Query List |
| --- | --- | --- | --- | --- | --- | --- | --- | --- | --- |
| BP | GO:0003300 | cardiac muscle hypertrophy | 6.373E-11 | 9.094E-8 | 4.067E-8 | 3.189E-7 | 5 | 134 | LMNA,EDN1,IGF1,TTN,NPPB |
| BP | GO:0014897 | striated muscle hypertrophy | 7.398E-11 | 1.056E-7 | 4.067E-8 | 3.189E-7 | 5 | 138 | LMNA,EDN1,IGF1,TTN,NPPB |
| BP | GO:0014896 | muscle hypertrophy | 8.550E-11 | 1.220E-7 | 4.067E-8 | 3.189E-7 | 5 | 142 | LMNA,EDN1,IGF1,TTN,NPPB |
| BP | GO:0048738 | cardiac muscle tissue development | 1.184E-8 | 1.689E-5 | 4.222E-6 | 3.311E-5 | 5 | 378 | LMNA,EDN1,IGF1,TTN,NPPB |
| BP | GO:0014706 | striated muscle tissue development | 1.591E-8 | 2.271E-5 | 4.541E-6 | 3.561E-5 | 5 | 401 | LMNA,EDN1,IGF1,TTN,NPPB |
| BP | GO:0055013 | cardiac muscle cell development | 3.461E-8 | 4.939E-5 | 8.232E-6 | 6.454E-5 | 4 | 145 | LMNA,EDN1,IGF1,TTN |
| BP | GO:0055006 | cardiac cell development | 4.891E-8 | 6.980E-5 | 8.792E-6 | 6.894E-5 | 4 | 158 | LMNA,EDN1,IGF1,TTN |
| BP | GO:0043500 | muscle adaptation | 5.146E-8 | 7.343E-5 | 8.792E-6 | 6.894E-5 | 4 | 160 | LMNA,EDN1,IGF1,NPPB |
| BP | GO:0060419 | heart growth | 5.545E-8 | 7.913E-5 | 8.792E-6 | 6.894E-5 | 4 | 163 | EDN1,IGF1,TTN,NPPB |
| BP | GO:0003012 | muscle system process | 8.086E-8 | 1.154E-4 | 1.154E-5 | 9.047E-5 | 5 | 555 | LMNA,EDN1,IGF1,TTN,NPPB |
| BP | GO:0055007 | cardiac muscle cell differentiation | 1.506E-7 | 2.149E-4 | 1.953E-5 | 1.532E-4 | 4 | 209 | LMNA,EDN1,IGF1,TTN |
| BP | GO:0009628 | response to abiotic stimulus | 1.787E-7 | 2.550E-4 | 2.125E-5 | 1.666E-4 | 6 | 1552 | LMNA,EDN1,IGF1,TTN,ACTB,NPPB |
| BP | GO:0060537 | muscle tissue development | 2.502E-7 | 3.571E-4 | 2.747E-5 | 2.154E-4 | 5 | 696 | LMNA,EDN1,IGF1,TTN,NPPB |
| BP | GO:0035051 | cardiocyte differentiation | 4.201E-7 | 5.995E-4 | 4.282E-5 | 3.358E-4 | 4 | 270 | LMNA,EDN1,IGF1,TTN |
| BP | GO:0035265 | organ growth | 4.722E-7 | 6.738E-4 | 4.492E-5 | 3.522E-4 | 4 | 278 | EDN1,IGF1,TTN,NPPB |
| BP | GO:0044057 | regulation of system process | 5.427E-7 | 7.744E-4 | 4.840E-5 | 3.795E-4 | 5 | 813 | LMNA,EDN1,IGF1,TTN,NPPB |
| BP | GO:0090257 | regulation of muscle system process | 9.145E-7 | 1.305E-3 | 7.676E-5 | 6.019E-4 | 4 | 328 | LMNA,EDN1,IGF1,TTN |
| BP | GO:0010611 | regulation of cardiac muscle hypertrophy | 1.695E-6 | 2.419E-3 | 1.288E-4 | 1.010E-3 | 3 | 92 | LMNA,EDN1,IGF1 |
| BP | GO:0055001 | muscle cell development | 1.715E-6 | 2.447E-3 | 1.288E-4 | 1.010E-3 | 4 | 384 | LMNA,EDN1,IGF1,TTN |
| BP | GO:0061061 | muscle structure development | 1.829E-6 | 2.610E-3 | 1.305E-4 | 1.023E-3 | 5 | 1038 | LMNA,EDN1,IGF1,TTN,ACTB |
| BP | GO:0014743 | regulation of muscle hypertrophy | 1.989E-6 | 2.839E-3 | 1.352E-4 | 1.060E-3 | 3 | 97 | LMNA,EDN1,IGF1 |
| BP | GO:0007507 | heart development | 2.196E-6 | 3.134E-3 | 1.425E-4 | 1.117E-3 | 5 | 1077 | LMNA,EDN1,IGF1,TTN,NPPB |
| BP | GO:0051047 | positive regulation of secretion | 3.489E-6 | 4.979E-3 | 2.165E-4 | 1.697E-3 | 4 | 459 | EDN1,IGF1,TTN,NPPB |
| BP | GO:0051050 | positive regulation of transport | 3.756E-6 | 5.359E-3 | 2.233E-4 | 1.751E-3 | 5 | 1200 | EDN1,IGF1,TTN,ACTB,NPPB |
| BP | GO:0043502 | regulation of muscle adaptation | 4.810E-6 | 6.863E-3 | 2.648E-4 | 2.076E-3 | 3 | 130 | LMNA,EDN1,IGF1 |

| Category | ID | Name | p-value | q-value Bonferroni | q-value FDR B&H | q-value FDR B&Y | Hit Count in Query List | Hit Count in Genome | Hit in Query List |
| --- | --- | --- | --- | --- | --- | --- | --- | --- | --- |
| CC | GO:0030133 | transport vesicle | 3.520E-4 | 4.857E-2 | 1.978E-2 | 1.090E-1 | 3 | 556 | EDN1,IGF1,NPPB |
| CC | GO:1990733 | titin-telethonin complex | 5.737E-4 | 7.917E-2 | 1.978E-2 | 1.090E-1 | 1 | 2 | TTN |
| CC | GO:0016363 | nuclear matrix | 6.935E-4 | 9.570E-2 | 1.978E-2 | 1.090E-1 | 2 | 144 | LMNA,ACTB |
| CC | GO:0034399 | nuclear periphery | 1.033E-3 | 1.426E-1 | 1.978E-2 | 1.090E-1 | 2 | 176 | LMNA,ACTB |
| CC | GO:0042567 | insulin-like growth factor ternary complex | 1.147E-3 | 1.583E-1 | 1.978E-2 | 1.090E-1 | 1 | 4 | IGF1 |
| CC | GO:0099013 | neuronal dense core vesicle lumen | 1.147E-3 | 1.583E-1 | 1.978E-2 | 1.090E-1 | 1 | 4 | IGF1 |
| CC | GO:0035867 | alphav-beta3 integrin-IGF-1-IGF1R complex | 1.147E-3 | 1.583E-1 | 1.978E-2 | 1.090E-1 | 1 | 4 | IGF1 |
| CC | GO:0098898 | dense core granule lumen | 1.147E-3 | 1.583E-1 | 1.978E-2 | 1.090E-1 | 1 | 4 | IGF1 |
| CC | GO:0016942 | insulin-like growth factor binding protein complex | 1.434E-3 | 1.978E-1 | 1.978E-2 | 1.090E-1 | 1 | 5 | IGF1 |
| CC | GO:0033093 | Weibel-Palade body | 1.720E-3 | 2.374E-1 | 1.978E-2 | 1.090E-1 | 1 | 6 | EDN1 |
| CC | GO:0005638 | lamin filament | 1.720E-3 | 2.374E-1 | 1.978E-2 | 1.090E-1 | 1 | 6 | LMNA |
| CC | GO:0048237 | rough endoplasmic reticulum lumen | 1.720E-3 | 2.374E-1 | 1.978E-2 | 1.090E-1 | 1 | 6 | EDN1 |
| CC | GO:0036454 | growth factor complex | 2.293E-3 | 3.164E-1 | 2.260E-2 | 1.245E-1 | 1 | 8 | IGF1 |
| CC | GO:0097433 | dense body | 2.293E-3 | 3.164E-1 | 2.260E-2 | 1.245E-1 | 1 | 8 | ACTB |
| CC | GO:0140092 | bBAF complex | 2.866E-3 | 3.955E-1 | 2.636E-2 | 1.452E-1 | 1 | 10 | ACTB |
| CC | GO:0000793 | condensed chromosome | 3.098E-3 | 4.276E-1 | 2.672E-2 | 1.472E-1 | 2 | 307 | TTN,ACTB |
| CC | GO:0071564 | npBAF complex | 4.010E-3 | 5.534E-1 | 2.845E-2 | 1.567E-1 | 1 | 14 | ACTB |
| CC | GO:0140288 | GBAF complex | 4.010E-3 | 5.534E-1 | 2.845E-2 | 1.567E-1 | 1 | 14 | ACTB |
| CC | GO:0035060 | brahma complex | 4.010E-3 | 5.534E-1 | 2.845E-2 | 1.567E-1 | 1 | 14 | ACTB |
| CC | GO:0005859 | muscle myosin complex | 4.582E-3 | 6.323E-1 | 2.845E-2 | 1.567E-1 | 1 | 16 | TTN |
| CC | GO:0098871 | postsynaptic actin cytoskeleton | 4.582E-3 | 6.323E-1 | 2.845E-2 | 1.567E-1 | 1 | 16 | ACTB |
| CC | GO:0016586 | RSC-type complex | 4.868E-3 | 6.717E-1 | 2.845E-2 | 1.567E-1 | 1 | 17 | ACTB |
| CC | GO:0071565 | nBAF complex | 4.868E-3 | 6.717E-1 | 2.845E-2 | 1.567E-1 | 1 | 17 | ACTB |
| CC | GO:0005614 | interstitial matrix | 5.153E-3 | 7.112E-1 | 2.845E-2 | 1.567E-1 | 1 | 18 | IGF1 |
| CC | GO:0005652 | nuclear lamina | 5.153E-3 | 7.112E-1 | 2.845E-2 | 1.567E-1 | 1 | 18 | LMNA |

| Pathway | M8728 | KEGG_HYPERTROPHIC_CARDIOMYOPATHY_HCM | KEGG Pathways | 1.787E-8 | 3.931E-7 | 2.731E-7 | 1.008E-6 | 4 | 83 | LMNA,IGF1,TTN,ACTB |
| --- | --- | --- | --- | --- | --- | --- | --- | --- | --- | --- |
| Pathway | M835 | KEGG_DILATED_CARDIOMYOPATHY | KEGG Pathways | 2.483E-8 | 5.462E-7 | 2.731E-7 | 1.008E-6 | 4 | 90 | LMNA,IGF1,TTN,ACTB |
| Pathway | M16376 | KEGG_ARRHYTHMOGENIC_RIGHT_VENTRICULAR_CARDIOMYOPATHY_ARVC | KEGG Pathways | 4.172E-4 | 9.178E-3 | 3.059E-3 | 1.129E-2 | 2 | 74 | LMNA,ACTB |
| Pathway | M7253 | KEGG_FOCAL_ADHESION | KEGG Pathways | 2.970E-3 | 6.534E-2 | 1.634E-2 | 6.029E-2 | 2 | 199 | IGF1,ACTB |
| Pathway | M16473 | KEGG_ALDOSTERONE_REGULATED_SODIUM_REABSORPTION | KEGG Pathways | 1.807E-2 | 3.976E-1 | 4.909E-2 | 1.812E-1 | 1 | 42 | IGF1 |
| Pathway | M7561 | KEGG_MTOR_SIGNALING_PATHWAY | KEGG Pathways | 2.234E-2 | 4.914E-1 | 4.909E-2 | 1.812E-1 | 1 | 52 | IGF1 |
| Pathway | M17906 | KEGG_VIBRIO_CHOLERAE_INFECTION | KEGG Pathways | 2.319E-2 | 5.101E-1 | 4.909E-2 | 1.812E-1 | 1 | 54 | ACTB |
| Pathway | M2333 | KEGG_PATHOGENIC_ESCHERICHIA_COLI_INFECTION | KEGG Pathways | 2.404E-2 | 5.288E-1 | 4.909E-2 | 1.812E-1 | 1 | 56 | ACTB |
| Pathway | M1835 | KEGG_GLIOMA | KEGG Pathways | 2.785E-2 | 6.128E-1 | 4.909E-2 | 1.812E-1 | 1 | 65 | IGF1 |
| Pathway | M6370 | KEGG_P53_SIGNALING_PATHWAY | KEGG Pathways | 2.912E-2 | 6.407E-1 | 4.909E-2 | 1.812E-1 | 1 | 68 | IGF1 |
| Pathway | M8232 | KEGG_LONG_TERM_DEPRESSION | KEGG Pathways | 2.997E-2 | 6.593E-1 | 4.909E-2 | 1.812E-1 | 1 | 70 | IGF1 |
| Pathway | M12294 | KEGG_VIRAL_MYOCARDITIS | KEGG Pathways | 2.997E-2 | 6.593E-1 | 4.909E-2 | 1.812E-1 | 1 | 70 | ACTB |
| Pathway | M15798 | KEGG_MELANOMA | KEGG Pathways | 3.039E-2 | 6.686E-1 | 4.909E-2 | 1.812E-1 | 1 | 71 | IGF1 |
| Pathway | M638 | KEGG_ADHERENS_JUNCTION | KEGG Pathways | 3.124E-2 | 6.872E-1 | 4.909E-2 | 1.812E-1 | 1 | 73 | ACTB |

| Disease | C0007193 | Cardiomyopathy, Dilated | DisGeNET Curated | 1.819E-6 | 6.384E-4 | 3.232E-4 | 2.082E-3 | 3 | 48 | LMNA,TTN,NPPB |
| --- | --- | --- | --- | --- | --- | --- | --- | --- | --- | --- |
| Disease | C1449563 | Cardiomyopathy, Familial Idiopathic | DisGeNET Curated | 2.060E-6 | 7.231E-4 | 3.232E-4 | 2.082E-3 | 3 | 50 | LMNA,TTN,NPPB |
| Disease | C1832931 | ARRHYTHMOGENIC RIGHT VENTRICULAR DYSPLASIA, FAMILIAL, 2 | DisGeNET Curated | 2.763E-6 | 9.697E-4 | 3.232E-4 | 2.082E-3 | 2 | 5 | LMNA,TTN |
| Disease | C1383860 | Cardiac Hypertrophy | DisGeNET Curated | 9.245E-6 | 3.245E-3 | 6.490E-4 | 4.179E-3 | 3 | 82 | EDN1,IGF1,NPPB |
| Disease | C0018800 | Cardiomegaly | DisGeNET Curated | 9.245E-6 | 3.245E-3 | 6.490E-4 | 4.179E-3 | 3 | 82 | EDN1,IGF1,NPPB |
| Disease | C0042514 | Tachycardia, Ventricular | DisGeNET Curated | 1.517E-5 | 5.325E-3 | 8.876E-4 | 5.715E-3 | 2 | 11 | LMNA,NPPB |
| Disease | C0878544 | Cardiomyopathies | DisGeNET Curated | 3.696E-5 | 1.297E-2 | 1.853E-3 | 1.193E-2 | 3 | 130 | EDN1,TTN,NPPB |
| Disease | C0376634 | Craniofacial Abnormalities | DisGeNET Curated | 6.375E-5 | 2.238E-2 | 2.011E-3 | 1.295E-2 | 3 | 156 | LMNA,EDN1,ACTB |
| Disease | C0235480 | Paroxysmal atrial fibrillation | DisGeNET Curated | 6.375E-5 | 2.238E-2 | 2.011E-3 | 1.295E-2 | 3 | 156 | EDN1,TTN,NPPB |
| Disease | C2585653 | Persistent atrial fibrillation | DisGeNET Curated | 6.375E-5 | 2.238E-2 | 2.011E-3 | 1.295E-2 | 3 | 156 | EDN1,TTN,NPPB |
| Disease | C3468561 | familial atrial fibrillation | DisGeNET Curated | 6.375E-5 | 2.238E-2 | 2.011E-3 | 1.295E-2 | 3 | 156 | EDN1,TTN,NPPB |
| Disease | C0004238 | Atrial Fibrillation | DisGeNET Curated | 6.875E-5 | 2.413E-2 | 2.011E-3 | 1.295E-2 | 3 | 160 | EDN1,TTN,NPPB |
| Disease | C0151744 | Myocardial Ischemia | DisGeNET Curated | 9.135E-5 | 3.206E-2 | 2.160E-3 | 1.391E-2 | 3 | 176 | EDN1,IGF1,ACTB |
| Disease | C0020538 | Hypertensive disease | DisGeNET Curated | 1.147E-4 | 4.027E-2 | 2.160E-3 | 1.391E-2 | 3 | 190 | EDN1,IGF1,NPPB |
| Disease | C0686353 | Muscular Dystrophies, Limb-Girdle | DisGeNET Curated | 1.361E-4 | 4.777E-2 | 2.160E-3 | 1.391E-2 | 2 | 32 | LMNA,TTN |
| Disease | C0238281 | Middle Cerebral Artery Syndrome | DisGeNET Curated | 1.538E-4 | 5.400E-2 | 2.160E-3 | 1.391E-2 | 2 | 34 | EDN1,IGF1 |
| Disease | C0751845 | Middle Cerebral Artery Embolus | DisGeNET Curated | 1.538E-4 | 5.400E-2 | 2.160E-3 | 1.391E-2 | 2 | 34 | EDN1,IGF1 |
| Disease | C0751847 | Embolic Infarction, Middle Cerebral Artery | DisGeNET Curated | 1.538E-4 | 5.400E-2 | 2.160E-3 | 1.391E-2 | 2 | 34 | EDN1,IGF1 |
| Disease | C0740391 | Middle Cerebral Artery Occlusion | DisGeNET Curated | 1.538E-4 | 5.400E-2 | 2.160E-3 | 1.391E-2 | 2 | 34 | EDN1,IGF1 |
| Disease | C0751846 | Left Middle Cerebral Artery Infarction | DisGeNET Curated | 1.538E-4 | 5.400E-2 | 2.160E-3 | 1.391E-2 | 2 | 34 | EDN1,IGF1 |
| Disease | C0740392 | Infarction, Middle Cerebral Artery | DisGeNET Curated | 1.538E-4 | 5.400E-2 | 2.160E-3 | 1.391E-2 | 2 | 34 | EDN1,IGF1 |
| Disease | C0751849 | Right Middle Cerebral Artery Infarction | DisGeNET Curated | 1.538E-4 | 5.400E-2 | 2.160E-3 | 1.391E-2 | 2 | 34 | EDN1,IGF1 |
| Disease | C0751848 | Thrombotic Infarction, Middle Cerebral Artery | DisGeNET Curated | 1.538E-4 | 5.400E-2 | 2.160E-3 | 1.391E-2 | 2 | 34 | EDN1,IGF1 |
| Disease | C0007222 | Cardiovascular Diseases | DisGeNET Curated | 1.727E-4 | 6.061E-2 | 2.245E-3 | 1.446E-2 | 2 | 36 | EDN1,NPPB |
| Disease | C0018798 | Congenital Heart Defects | DisGeNET Curated | 2.588E-4 | 9.082E-2 | 2.247E-3 | 1.447E-2 | 2 | 44 | EDN1,NPPB |
| Disease | C0020649 | Hypotension | DisGeNET Curated | 3.761E-4 | 1.320E-1 | 2.247E-3 | 1.447E-2 | 2 | 53 | EDN1,NPPB |
| Disease | 603689 | Myopathy, Myofıbrıllar, 9, Wıth Early Respıratory Faılure | OMIM MedGen | 5.763E-4 | 2.023E-1 | 2.247E-3 | 1.447E-2 | 1 | 1 | TTN |
| Disease | C0037188 | Sinoatrial Block | DisGeNET Curated | 5.763E-4 | 2.023E-1 | 2.247E-3 | 1.447E-2 | 1 | 1 | LMNA |
| Disease | C0242707 | Right Ventricular Dysfunction | DisGeNET Curated | 5.763E-4 | 2.023E-1 | 2.247E-3 | 1.447E-2 | 1 | 1 | NPPB |
| Disease | C1857829 | Heart-hand syndrome, Slovenian type | DisGeNET Curated | 5.763E-4 | 2.023E-1 | 2.247E-3 | 1.447E-2 | 1 | 1 | LMNA |
| Disease | C2673677 | Myopathy, Early-Onset, with Fatal Cardiomyopathy | DisGeNET Curated | 5.763E-4 | 2.023E-1 | 2.247E-3 | 1.447E-2 | 1 | 1 | TTN |
| Disease | 613765 | CARDIOMYOPATHY, FAMILIAL HYPERTROPHIC, 9 | OMIM MedGen | 5.763E-4 | 2.023E-1 | 2.247E-3 | 1.447E-2 | 1 | 1 | TTN |
| Disease | 610140 | HEART-HAND SYNDROME, SLOVENIAN TYPE | OMIM MedGen | 5.763E-4 | 2.023E-1 | 2.247E-3 | 1.447E-2 | 1 | 1 | LMNA |
| Disease | 608747 | INSULIN-LIKE GROWTH FACTOR I DEFICIENCY | OMIM MedGen | 5.763E-4 | 2.023E-1 | 2.247E-3 | 1.447E-2 | 1 | 1 | IGF1 |
| Disease | 604145 | CARDIOMYOPATHY, DILATED, 1G | OMIM MedGen | 5.763E-4 | 2.023E-1 | 2.247E-3 | 1.447E-2 | 1 | 1 | TTN |
| Disease | C0085128 | Cardiac Output, High | DisGeNET Curated | 5.763E-4 | 2.023E-1 | 2.247E-3 | 1.447E-2 | 1 | 1 | NPPB |
| Disease | C1861065 | CARDIOMYOPATHY, FAMILIAL HYPERTROPHIC, 9 | DisGeNET Curated | 5.763E-4 | 2.023E-1 | 2.247E-3 | 1.447E-2 | 1 | 1 | TTN |
| Disease | C1863599 | Hereditary Myopathy with Early Respiratory Failure | DisGeNET Curated | 5.763E-4 | 2.023E-1 | 2.247E-3 | 1.447E-2 | 1 | 1 | TTN |
| Disease | 115200 | CARDIOMYOPATHY, DILATED, 1A | OMIM MedGen | 5.763E-4 | 2.023E-1 | 2.247E-3 | 1.447E-2 | 1 | 1 | LMNA |
| Disease | C1858763 | Cardiomyopathy, Dilated, 1g | DisGeNET Curated | 5.763E-4 | 2.023E-1 | 2.247E-3 | 1.447E-2 | 1 | 1 | TTN |
| Disease | 212112 | CARDIOMYOPATHY, DILATED, WITH HYPERGONADOTROPIC HYPOGONADISM | OMIM MedGen | 5.763E-4 | 2.023E-1 | 2.247E-3 | 1.447E-2 | 1 | 1 | LMNA |
| Disease | C0033141 | Cardiomyopathies, Primary | DisGeNET Curated | 6.376E-4 | 2.238E-1 | 2.433E-3 | 1.566E-2 | 2 | 69 | EDN1,NPPB |
| Disease | C0036529 | Myocardial Diseases, Secondary | DisGeNET Curated | 6.376E-4 | 2.238E-1 | 2.433E-3 | 1.566E-2 | 2 | 69 | EDN1,NPPB |
| Disease | C0004245 | Atrioventricular Block | DisGeNET Curated | 1.152E-3 | 4.042E-1 | 3.485E-3 | 2.244E-2 | 1 | 2 | LMNA |
| Disease | C0023212 | Left-Sided Heart Failure | DisGeNET Curated | 1.612E-3 | 5.659E-1 | 4.421E-3 | 2.847E-2 | 2 | 110 | EDN1,NPPB |
| Disease | C1959583 | Myocardial Failure | DisGeNET Curated | 1.612E-3 | 5.659E-1 | 4.421E-3 | 2.847E-2 | 2 | 110 | EDN1,NPPB |
| Disease | C0018802 | Congestive heart failure | DisGeNET Curated | 1.612E-3 | 5.659E-1 | 4.421E-3 | 2.847E-2 | 2 | 110 | EDN1,NPPB |
| Disease | C0018801 | Heart failure | DisGeNET Curated | 1.612E-3 | 5.659E-1 | 4.421E-3 | 2.847E-2 | 2 | 110 | EDN1,NPPB |
| Disease | C1961112 | Heart Decompensation | DisGeNET Curated | 1.612E-3 | 5.659E-1 | 4.421E-3 | 2.847E-2 | 2 | 110 | EDN1,NPPB |
| Disease | C0018794 | Heart Block | DisGeNET Curated | 2.302E-3 | 8.081E-1 | 5.535E-3 | 3.564E-2 | 1 | 4 | LMNA |
| Disease | C0878773 | Overactive Bladder | DisGeNET Curated | 2.302E-3 | 8.081E-1 | 5.535E-3 | 3.564E-2 | 1 | 4 | EDN1 |
| Disease | C0004331 | Auriculo-Ventricular Dissociation | DisGeNET Curated | 2.302E-3 | 8.081E-1 | 5.535E-3 | 3.564E-2 | 1 | 4 | LMNA |
| Disease | C0410204 | Myopathy, Centronuclear, Autosomal Recessive | DisGeNET Curated | 2.302E-3 | 8.081E-1 | 5.535E-3 | 3.564E-2 | 1 | 4 | TTN |
| Disease | C0221054 | Welander Distal Myopathy | DisGeNET Curated | 2.302E-3 | 8.081E-1 | 5.535E-3 | 3.564E-2 | 1 | 4 | TTN |
| Disease | C0011644 | Scleroderma | DisGeNET Curated | 2.302E-3 | 8.081E-1 | 5.535E-3 | 3.564E-2 | 1 | 4 | LMNA |
| Disease | C0016508 | Congenital Foot Deformity | DisGeNET Curated | 2.302E-3 | 8.081E-1 | 5.535E-3 | 3.564E-2 | 1 | 4 | LMNA |
